# Health care pathways described by care-seekers following a call to the emergency medical communication center—A Swedish perspective

**DOI:** 10.1371/journal.pone.0325706

**Published:** 2025-06-13

**Authors:** Jonas Wihlborg, Anna Carin Wahlberg, Mats Holmberg, Lars Sturesson, Annika Alm-Pfrunder, Veronica Lindström

**Affiliations:** 1 School of Health and Welfare, Dalarna University, Falun, Sweden; 2 Department of Neurobiology, Care Sciences and Society, Section of Nursing, Karolinska Institutet, Stockholm, Sweden; 3 Faculty of Health and Life Sciences, Linnaeus University, Växjö, Sweden; 4 Centre for Clinical Research Sörmland, Uppsala University, Eskilstuna, Sweden; 5 Department of Ambulance Service, Region Sörmland, Katrineholm, Sweden; 6 Department of Nursing, Division of Ambulance service, Region Västerbotten, Umeå University Umeå, Umeå, Sweden; Jazan University College of Applied Medical Science, SAUDI ARABIA

## Abstract

**Introduction:**

A call to an emergency number is often the first course of action among care-seekers seeking emergency care. The emergency medical communication centre assesses the call and dispatches resources to meet the care-seekers’ care needs. Knowledge concerning specific healthcare pathways is sparse and does not include the care-seeker perspective. Therefore, the aim of this study was to explore care-seekers’ experiences of healthcare pathways following a call to the national emergency number.

**Methods:**

The study was an explorative cross-sectional study using data from telephone interviews with callers to the Swedish national emergency number. Study informants (n = 141) provided descriptions of incidents (n = 156) that led up to the call, as well as their experiences following a healthcare pathway. Data analysis included mapping healthcare pathways, descriptive statistics, and gathering descriptions of care-seeker experiences.

**Results and conclusions:**

This study shows the multiple healthcare pathways for care-seekers following a call to the national emergency number. The accessibility and availability of the healthcare system are found to be difficult to grasp among care-seekers. Decisions on which pathway to follow are primarily made by healthcare professionals rather than the care-seekers, which supports the notion that the system is not being designed from the care-seekers’ perspective. Expressions of increased vulnerability, being unsafe and feeling lost were common among the care-seekers, regardless of which pathway was followed. The findings of this study could be used as an incentive for healthcare providers to develop a healthcare system based on the found novel knowledge of the care-seekers’ perspective.

## Introduction

Access to emergency care is central in the healthcare system and is defined by Levesque et al. as *“the opportunity to reach and obtain appropriate healthcare services in situations of perceived need for care*” (p. 4) [1]. When emergency care is needed, the emergency medical services (EMS) system gives access and delivers emergency care to care-seekers. EMSs differ worldwide, but regardless of system, the patient’s journey in the EMS is similar in urban countries. After an acute illness, injury, or incident, a call is made to an emergency number. Call-takers at the Emergency Medical Communication Centre (EMCC) assess the call and provide medical or other advice to the caller [2,3] or dispatch the appropriate resources [4]. It is known that call-takers working with different kinds of telephone triage and/or consultations have an important function in emergencies and out-of-hours care [5]. However, triaging and consultations through phone calls are complex since the call-taker’s primary source of information is the caller’s verbal descriptions, descriptions that may be hindered by communication barriers [6–9] and/or lack of information [10]. The overall implication is that the outcome of the call to an EMCC or other healthcare advice centres depends on the caller’s ability to describe the experienced condition and symptoms, as well as the call-taker’s ability to interpret the caller’s information [11].

The incidence of calling the emergency number is reported to be 60/1000 citizens/per year in Denmark [12], but whether this is true for most countries is not known. However, callers have expressed concerns about calling an emergency number, questioning whether their call is necessary and whether their symptoms are indeed acute [13–15]. However, after deciding to call the emergency number, it has been reported that callers may experience a long wait time, and while waiting, some callers may feel vulnerable, lonely, fearful, and dependent on receiving help. Despite different experiences, the caller is usually satisfied with the EMCC [13,16], and it has also been described that contact with the call-taker at the EMCC can be a lifeline [16]. The time between the emergency call and the arrival of dispatched resources at the care-seeker’s location has been described as exhausting for the care-seeker [15,17]. Still, if callers were provided with information about the estimated time of arrival or were given a task, such as packing a bag or opening doors, they felt calmer [15]. When the dispatched resources, usually an ambulance, arrive at the scene, an assessment is made, symptom alleviation is initiated, and commonly, if needed, a transport is conducted to the appropriate level of care [4]. However, when no resource is dispatched by the EMCC, or if the care-seeker is non-conveyed by the ambulance personnel, the care-seeker’s are often left in uncertainty of what happens after the care encounter. The care-seekers are generally satisfied with the care provided by the ambulance service [18,19] but being non-conveyed is also connected to emotions such as fear [20], which could be an explanation for why more than half (53%) of the non-conveyed care-seekers sought care again within 72 hours [21]. Nevertheless, if the knowledge is limited regarding the non-conveyed care-seekers, the understanding of what happens to the care-seekers after an emergency call is an unexplored area. There is limited research on pathways the care-seeker gets through in the chain of emergency care, and the existing research does not provide a comprehensive understanding of the healthcare process from the care-seeker’s point of view. Therefore, the aim of this study was to describe care-seekers’ experiences of their healthcare pathways following a call to the emergency number.

## Methods

The study had an explorative design using data gathered from telephone interviews with callers to the emergency number. The study conforms to the Strengthening the Reporting of Observational Studies in Epidemiology (STROBE) cross-sectional reporting guidelines [22].

### Settings

In Sweden, the emergency number 112 is operated by SOS Alarm, a Swedish publicly owned company. To ensure coordinated actions by EMS, the police, and the fire brigade, all emergency calls to 112 are received by call-takers at an EMCC. During the study period, 16 EMCCs in different geographic locations answered approximately 3.3 million calls, and one-third of these calls concerned medical complaints or care assignments [3]. According to national regulations, the response time for a 112 call should not exceed eight seconds, and when the time is exceeded, the call is transferred to another EMCC. Consequently, sometimes a caller from southern Sweden can speak with a call-taker in northern Sweden. The call-taker is someone with or without medical education who undergoes six months of training before certification, and a re-certification is required annually. In some EMCCs, physicians and registered nurses are also available for medical consultations. In the emergency call, the call-taker must first determine whether vital signs of the care-seeker (e.g., consciousness, breathing) are present and the location. Thereafter, the call-taker asks the caller questions about the incident, the care-seeker’s signs and symptoms and previous medical history. To support the call-takers at SOS Alarm with the assessment and prioritisation of calls, a Swedish medical emergency index was used during the study period. The Swedish Medical Index is criteria-based and consists of 34 main chapters divided into priority levels based on symptom presentation. Each chapter provides call-takers with a series of questions to ask the caller [23]. After assessment, the call-taker’s actions may include dispatching an ambulance, referring the patient to alternative transportation, or providing instructions to the callers to contact other healthcare facilities.

### Procedures

**Fig 1:**
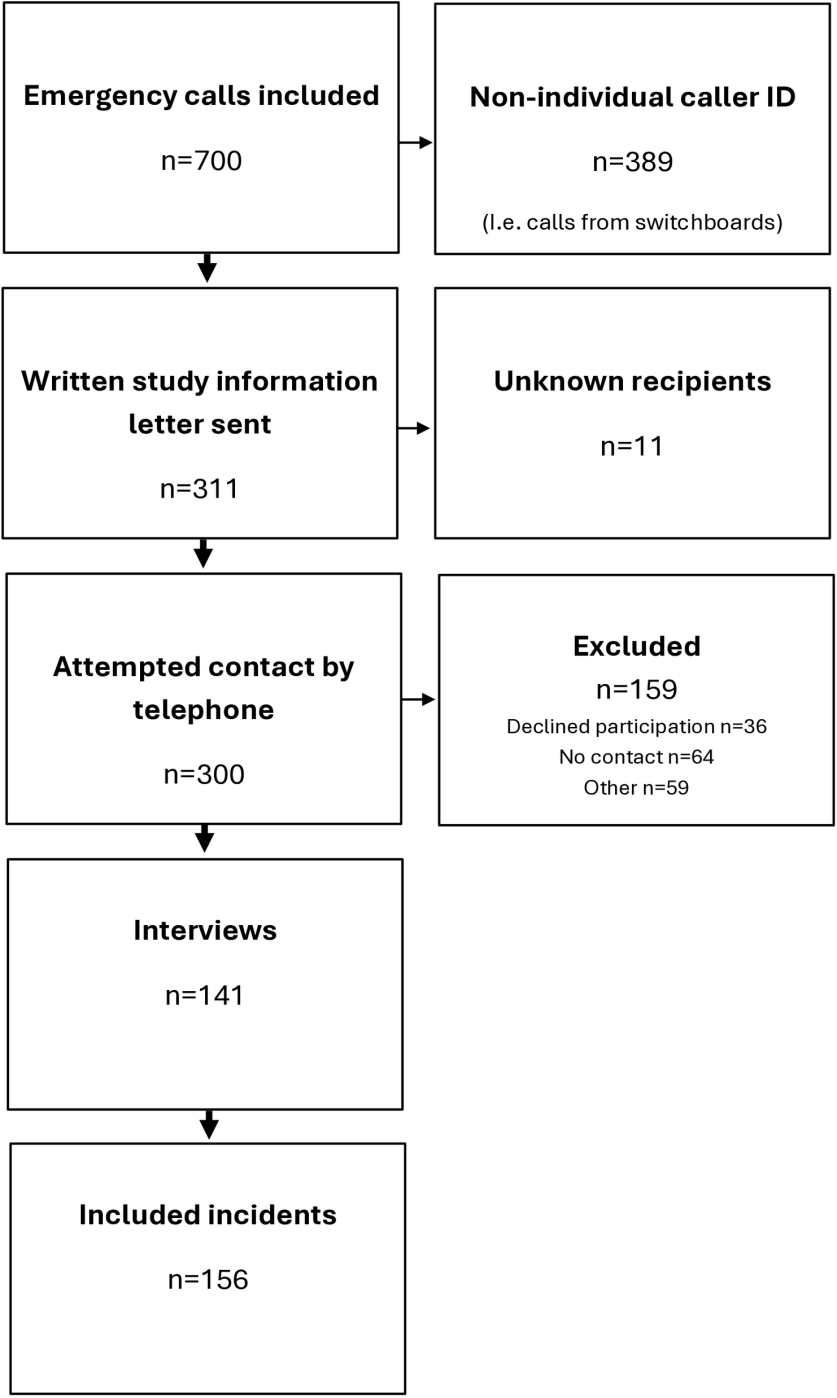
Overview of recruitment and data collection procedures.

Study participants were recruited by using caller information collected and shared by one EMCC in Sweden. To ensure a random selection of participants as well as varied sample of data in accordance with the study aim, caller information from calls made to EMCCs classified as care assignments by the EMCC was used. Phone numbers from the first three care assignment calls every hour were collected and were identified as eligible for study inclusion. The eligible calls (n = 700) for inclusion were evenly distributed over all hours of the day on all weekdays during the period 12^th^ of August to the 29^th^ of September 2019. Calls from non-identifiable callers were excluded in the next step (Fig 1). Excluded calls were made from non-individual numbers, such as unregistered numbers, company-registered phones, via switchboards, or hidden numbers (n = 389). Names and postal addresses associated with the caller’s telephone number were retrieved from a national search engine and used to send written study information before potential study inclusion (n = 311). Eleven of the letters were returned unopened by the postal service due to wrong or unknown addresses or unknown recipients. The authors made up to three attempts to contact the remaining callers by phone one week after the information letter was sent (n = 300). A total of just over 20 percent (20.1%) of the original calls agreed to further inclusion in the study (n = 141). The excluded callers at this stage were unreachable by phone (n = 64), declined study participation (n = 36), or provided other reasons for exclusion (n = 59). Reasons for the exclusion of calls at this stage were that callers provided no explanation, declared they had not made calls, or had language, hearing, cognitive or communicative problems.

### Data collection

Data was gathered by telephone interviews (n = 141). The interview began with the question, “*Can you describe why you called 112?”* This was followed by the question, “What happened within 72 hours after the call?” In addition, questions were asked about actions taken by the EMCC, care pathways, care given, and characteristics of the EMCC callers and care seekers. The average interview time was 10 minutes per interview, with a range of 4–46 minutes. All interviews were audio-recorded, and data was manually extracted and transferred by each author to a results matrix sheet. The result matrix sheet was divided into three categories, where the first consisted of statements about actions taken by the dispatch centre, care-seeker pathway and care given within 72 hours of the EMCC call. The second partition included characteristics of the EMCC callers and care-seekers as well as the reasons initially leading to the call to the emergency number. The third partition comprised a summary of callers’ experiences 72 hours after calling the emergency number. Nine of the participants provided more than one description of an emergency call, which rendered 156 calls to be analysed.

### Participants

All study participants had experiences of making one or more emergency calls to the EMCC during the data collection period from which the study data were extracted through the study interview. In this study, “care-seekers” is used as an overarching term, encompassing not only the person in need of care but also all study informants who provided data that contributed to the results.

### Analysis

Descriptive data on care-seekers, callers and reasons for calling were summarised and presented using descriptive statistics. Data gathered on actions, pathways and care given from the reported calls (n = 156) were collected. To further illustrate the differences in how participants described healthcare pathways and emotions, examples are presented in the results section after ensuring the anonymisation of the callers’ descriptions.

### Ethics approval and consent to participate

The study was vetted and approved by the Regional Ethical Review Board in Stockholm, Sweden (Reference No: 2018/1831-31/5). Informed consent was obtained from all subjects and/or their legal guardian(s). Written study information was provided to the participants by mail one week ahead of attempted study inclusion, allowing the informants to make an informed decision on participation ahead of study inclusion. Consent to participate was obtained orally and audio recorded at the start of the interview by each interviewing author.

## Results

### The calls

The reason for calling the EMCC varied among the callers, even though all emergency calls were classified as care assignments by the EMCC. The reported types of reasons leading up to the emergency call included a variety of accidents, trauma and illness observed by the callers with most of the individual care-seeker origin. All included EMCC calls were made by adult callers (aged 20–89 years), with the majority being female callers (61%). The care-seekers consisted predominantly of elderly persons (Md = 73) with an even gender distribution, as shown in Table 1. The majority of EMCC calls (66%) were made by the care-seekers’ significant others rather than by the care-seekers themselves. Bystanders with no prior personal connection to the care-seekers made up the second largest group of callers (14%), while the care-seekers themselves made fewer (8%) calls. Some of the emergency calls (9%) were the result of an assessment of care needs by healthcare professionals following professional contact between them and the care-seekers (Table 1).

**Table 1:** Characteristics of EMCC callers and care-seekers.

	Incidents (n = 156)
**Caller Gender**	
Men	39% (61)
Women	61% (95)
**Caller Age**, Mean (years) ± SD¹	61 ± 18
Median	63
Min-Max	20-89
**Caller**	
Significant other	66% (103)
Bystander	14% (23)
Healthcare professional	9% (14)
Care seeker Missing	8% (12) 3% (4)
**Care seeker Gender**	
Men	46% (72)
Women	54% (84)
**Care seeker Age**, Mean (years) ± SD^¹^ Median Min-Max	67 ± 21 73 9-99
**Incident type**	
Fall	13% (21)
Unconsciousness	13% (21)
Infection	8% (12)
Abdominal pain	7% (11)
Poor general condition	7% (11)
Stroke	7% (11)
Chest pain	6% (10)
Breathing difficulties	5% (9)
Pain	5% (9)
Dizziness	4% (7)
Seizure	4% (7)
Other	4% (7)
Bleeding	3% (5)
Mental illness	3% (5)
Allergic reaction	2% (4)
Traffic accident	2% (3)
Arrhythmia	2% (3)

¹ *Standard deviation.*

**Fig 2:**
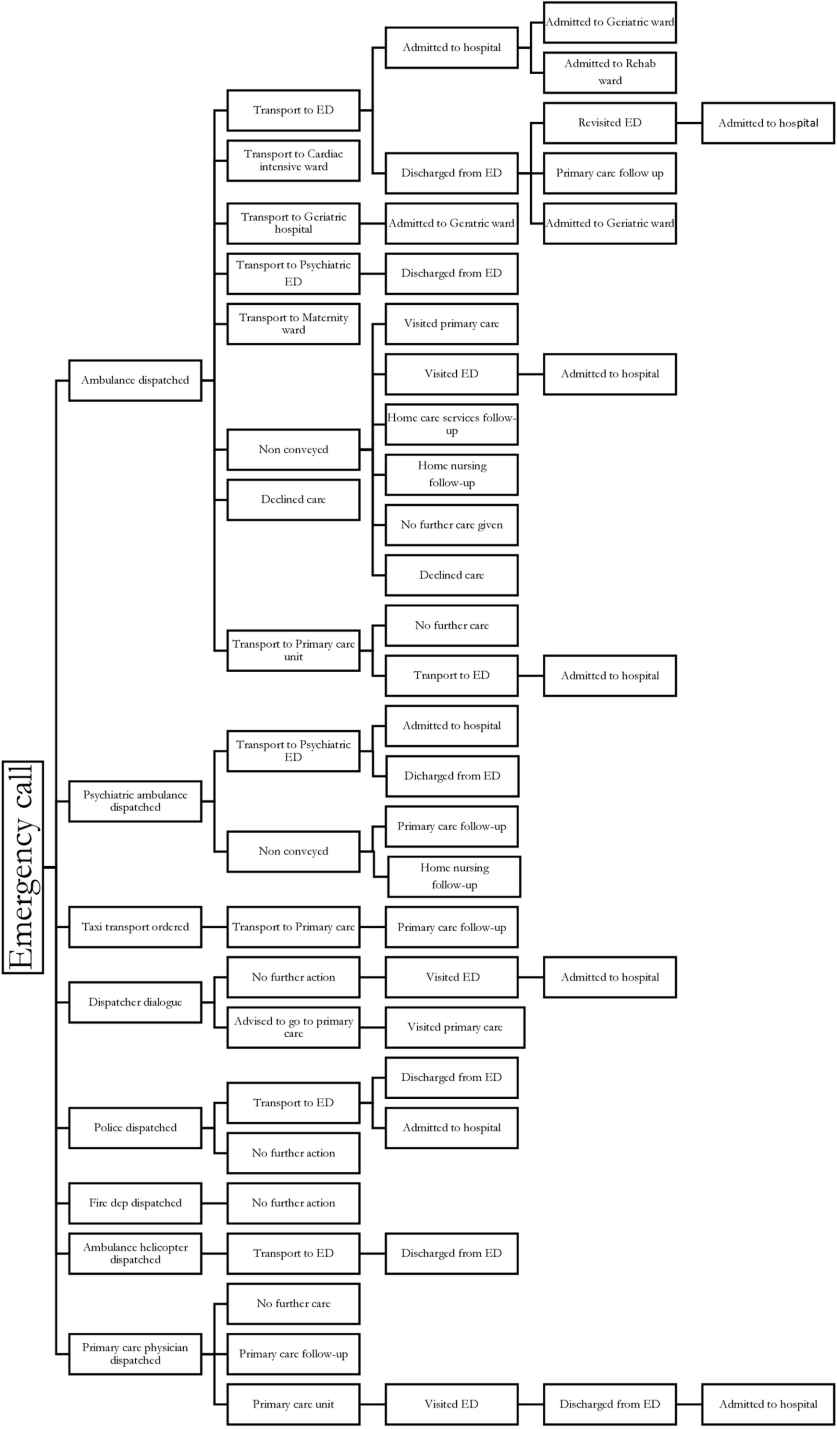
Emergency call actions and pathways.

Each emergency call classified as a care assignment triggered a call-taker action to respond to the call and the care-seeker’s need for care. The actions taken varied from problem-solving through dialogue between the caller and call-taker to dispatching one or more units to reach the care-seeker at the place of the incident or illness. The actions identified in this study included: call-taker dialogue only, ordering a taxi transport or dispatch of EMS including ambulance services, psychiatric ambulance services, ambulance helicopter, or primary care physician, police department or fire department units (Fig 2).

As each call was subject to a decision regarding the care-seeker’s need of care, the decision led to various possible pathways and outcomes for the caller within 72 hours from the emergency call, as shown in Fig 2. Examples of pathways identified in this study after the emergency call are: ambulance – transport to Emergency Department (ED) – discharged from ED – revisiting ED- and admission to an hospital ward; ambulance – direct admission to geriatric hospital or other specialist wards at the hospital; non-conveyance by the ambulance, sometimes including follow-up by other care givers; primary care physicians dispatched – referral to primary care unit – referral to ED – discharged from ED – admission to a hospital ward. The most common pathway involved ambulance dispatch and transport to the ED (82%), and 26% of those care-seekers were discharged from the ED within 8 hours. Non-conveyance decisions made by the ambulance personnel was 11%. The pathways that included different levels of care, culminating in a hospital admission, accounted for 6% of the total number of incidents.

### Descriptions of healthcare pathways for care-seekers following a call to the emergency medical communication centre

In the interviews, some callers described having called the national healthcare call centre, 1177, for healthcare advice and information before calling the emergency number. The callers who called the emergency number and were advised by the registered nurses at 1177 described how they had to reiterate their symptoms, situation, and medical history, as there was no communication between the call-taker in the EMCC and the nurses at 1177. The need to reiterate information when calling the emergency number was sometimes perceived as burdensome by the callers, they also became uncertain about what they had said to whom.

A common healthcare pathway after the emergency number was when an ambulance was dispatched, and the care-seeker was taken to the ED by an ambulance. Some of the care-seekers were admitted, and some were sent home from the ED within 24 hours and returned to the ED within 72 hours and then were admitted to a hospital ward. In these situations, some of the care-seekers were reasoning about why they were not admitted to a ward the first time they came to the ED. An example of when an ambulance was dispatched: a call from the daughter of an 83-year-old woman who had fallen and was bleeding from the head. The ambulance personnel made an assessment and then left the elderly woman in the care of the daughter, advising her to contact her mother’s regular physician. The woman or daughter did not receive any follow-up from the EMCC or the ambulance services, and they did not follow the advice given by the ambulance personnel. Other callers reported that after an assessment by ambulance personnel, they received self-care advice, for example, taking fever-reducing medication regularly, using rehydration solution, and, in the event of deterioration or new symptoms, contacting 1177 or the emergency number. Some called 1177 or the emergency number, someone went to the ED by their own car, and some care-seekers required no further care contacts within 72 hours after the first emergency call. Sometimes, the call-takers and/or ambulance personnel’s recommendation was to contact their primary healthcare centre. The care-seekers followed the recommendation, and some were then referred to the ED by their primary healthcare physician. Another caller said that the ambulance transported her to the ED, where no action was taken, and no information was given as perceived by the caller; instead, the care-seeker visited the primary healthcare centre on her own initiative. One example of when no ambulance was dispatched was the case of a parent who called about their child with suspected fever convulsions and was asked to wait and see what happened. The parents received a follow-up call from the EMCC and stayed home with no further actions taken by healthcare personnel. In a call where no action was taken by the call-taker at the EMCC, a woman said that she transported herself to the ED and was admitted to the hospital for 36 hours and then was discharged home with self-care advice. Another caller described how they tried with no success to contact their ordinary caregiver, resulting in an emergency call and transport to the ED instead of direct admission to a specialist ward.

In another call, a 60-year-old woman asked her home care personnel to call the emergency number, and a psychiatric ambulance visited the woman. After a one-hour visit, the ambulance left. The home care staff visited the woman as usual afterwards, but no further action was taken by the psychiatric ambulance or EMCC as perceived by the woman. When an ambulance, police and the fire brigade were dispatched, it was usually because of an accident, and the injured person was transported to the ED by ambulance or, in some cases, by the police. The outcome at the ED was either observation for a couple of hours or hospitalisation for more than 72 hours. A primary care physician was dispatched to a care-seeker after assessment by the call-taker at the EMCC. The patient had pain in their foot and was recommended by the physician to visit a primary care unit. The primary care physician referred the patient to the ED which sent the patient home with a referral to surgery.

During the interviews the callers described a range of emotions concerning the outcome of the emergency call: relief at staying home after the ambulance personnel made the assessment; frustration after several referrals to different levels of care; anger at not receiving the care expected; loneliness to be left home alone; a sense of safety when being taken care of; or a sense of safety when receiving a follow-up call but also feelings of being lost about what to do as a next step, and a determination to get help. The callers also described appreciation towards the interviewers following the interview call, thus having a possibility to share their experiences like they would in a follow-up call.

## Discussion

The overall findings of this study are that healthcare pathways for the care-seekers after calling the emergency number are not straightforward. As described by Levesque et al. [1], this may imply that the availability of care is difficult to identify by both care-seekers and healthcare professionals. It may also imply that the availability of healthcare is organised from the healthcare professionals’ perspectives and does not reflect the care-seekers’ needs. However, access to healthcare, including emergency care, is complex and influenced by several internal and external factors such as health literacy, health beliefs, geographic location, hours of opening, technical and interpersonal quality and financial resources [1]. Previous studies have shown that when deciding to call the emergency number, the callers experience emotions such as vulnerability, loneliness, fear and being dependent on receiving help [13,16]. In this study, similar emotions were expressed by the care-seekers, but they also expressed frustration and anger at being referred between health-care facilities. The negative emotions described by the participants can be attributed to a lack of knowledge among callers about various healthcare options and the limited availability of healthcare facilities. A reason for referring care-seekers could be that the call-takers at the EMCC are acting as gatekeepers for the ambulance service and potentially for hospital admissions, as described in previous studies [12,24]. Regardless of potential gatekeepers, the care-seekers are in a vulnerable situation when seeking care for illnesses and/or injuries and being vulnerable and refused care may cause an increase in suffering for the patients. This calls for further research to determine how to support care-seekers in the chain of perceived emergency care. The emotions described by the care-seekers may also be an expression of their expectation of being offered care that is appropriate to their perceived need for care. The care-seekers had received access to the EMS by calling the emergency number, but when they did not receive care from healthcare professionals as expected, they faced a different perception of the care needed. Considering this, the care-seekers’ experiences of calling the emergency number and what happens within 72 hours after the call may represent a crucial time frame where barriers to accessing emergency care can be identified. It has previously been discussed that the call-takers’ decision-making process around how to respond to the call could be improved by incorporating care-seekers’ interpretation of the severity of the experienced illness [25,26]. Whether that is true is not clear from this study’s findings, but it may be true that the care-seekers have the best knowledge about their illness. However, further research is needed to determine the most effective way of incorporating care-seekers into the assessment of resources required when they have made an emergency call. Six percent of the care-seekers were admitted to the hospital after being assessed by multiple healthcare professionals following the emergency call, and theoretically, this may have posed a patient safety risk. Within the ambulance service, it is reported that approximately 1 in 10 patients are at risk of exposure to patient safety risks, and a median of 15% suffer harm [27]. Based on the findings of this study, it is unclear whether any patients experienced harm; however, it is reasonable to assume that care seekers are at risk of suffering healthcare-related harm when being referred between different healthcare providers.

## Limitations

This study´s aim was to explore care-seekers’ descriptions of their healthcare pathways, but in doing so, the frequencies of pathway usage were intentionally left briefly described. Information on frequencies of pathway usage could perhaps be considered as adding to the description of the phenomenon, which could increase the validity of the results. However, this was considered as another research aim which could benefit from another study design and setting. Another possible weakness of the study is the reliability of the data. The first step in data collection was the recognition of callers from calls made to EMCC that were classified as care assignments. This recognition was logged and stored in the EMCC data recording system where the information used for data collection was gathered. However, if the call-taker redirects a call or fails to recognise it as a care assignment, the classification is not carried out, and the caller’s information is not registered. Hence, the assumption that there could be a group of unclassified calls emphasises the view of uncertainty regarding the possibility of this study to provide a complete picture of pathways. Regardless of the unclassified calls, we value that the group investigated is, to some extent, representative, as the percentage of care-seekers non-conveyed by ambulance personnel is comparable to previous studies on non-conveyance conducted in Sweden [28,29]. Furthermore, our callers’ characteristics are similar compared with previous studies [30]. Study exclusion criteria for callers, including language, hearing, cognitive, or communicative problems, may have affected the representativeness of the results, potentially underestimating the actual complexity of the problems described. This should be considered when interpreting the results. The validity of the study data could also be considered as some of the informants expressed uncertainty regarding the events during the 72-hour period. Some informants referred to prior or later events or had problems recalling the actual events. Also, missing data is reportedly large in this study due to the measures and outcome being unknown by bystanders and healthcare professional callers. However, the validity of the results was strengthened by the amount of interviews.

## Conclusions

This study shows the multiple healthcare pathways for care-seekers following a call to the EMCC. The accessibility and the availability of the healthcare system is found to be complex and hard to fully understand among the care-seekers and maybe also for the healthcare professionals. Decisions on which pathway to follow are mainly made by healthcare professionals rather than the care-seekers, which adds to the notion that the system is not designed from the care-seekers’ perspective. The transition between caregivers is especially challenging and aggravated by lack of joint information systems. Expressions of increased vulnerability, being unsafe and feeling lost were common among the care-seekers, regardless of which pathway was followed. The findings of this study could be used as an incentive to healthcare providers to develop a healthcare system based on the care-seekers’ perspective.

## References

[1] Levesque J-F, Harris MF, Russell G. Patient-centred access to health care: conceptualising access at the interface of health systems and populations. Int J Equity Health. 2013;12:18. doi: 10.1186/1475-9276-12-18 23496984 PMC3610159

[2] Lindström V, Bohm K, Kurland L. Prehospital care in Sweden. Notfall Rettungsmed. 2015;18(2):107–9. doi: 10.1007/s10049-015-1989-1

[3] SOS Alarm, Sustainability and Annual Report. Stockholm, Sweden. 2019. https://www.sosalarm.se/contentassets/56c42981f0b9491697f54ce0707a7ebd/sos-alarm---arsberattelse-2019.pdf

[4] Castrén M, Karlsten R, Lippert F, Christensen EF, Bovim E, Kvam AM, et al. Recommended guidelines for reporting on emergency medical dispatch when conducting research in emergency medicine: the Utstein style. Resuscitation. 2008;79(2):193–7. doi: 10.1016/j.resuscitation.2008.07.007 18805620

[5] Huibers L, Smits M, Renaud V, Giesen P, Wensing M. Safety of telephone triage in out-of-hours care: a systematic review. Scand J Prim Health Care. 2011;29(4):198–209. doi: 10.3109/02813432.2011.629150 22126218 PMC3308461

[6] Grow RW, Sztajnkrycer MD, Moore BR. Language barriers as a reported cause of prehospital care delay in Minnesota. Prehosp Emerg Care. 2008;12(1):76–9. doi: 10.1080/10903120701709878 18189182

[7] Holmström IK, Kaminsky E, Lindberg Y, Spangler D, Winblad U. The perspectives of Swedish registered nurses about managing difficult calls to emergency medical dispatch centres: a qualitative descriptive study. BMC Nurs. 2021;20(1):150. doi: 10.1186/s12912-021-00657-5 34407818 PMC8371756

[8] Wahlberg AC, Cedersund E, Wredling R. Telephone nurses’ experience of problems with telephone advice in Sweden. J Clin Nurs. 2003;12(1):37–45. doi: 10.1046/j.1365-2702.2003.00702.x 12519248

[9] Wahlberg AC, Cedersund E, Wredling R. Factors and circumstances related to complaints in emergency medical dispatching in Sweden: an exploratory study. Eur J Emerg Med. 2003;10(4):272–8. doi: 10.1097/00063110-200312000-00006 14676503

[10] Lindström V, Heikkilä K, Bohm K, Castrèn M, Falk A-C. Barriers and opportunities in assessing calls to emergency medical communication centre--a qualitative study. Scand J Trauma Resusc Emerg Med. 2014;22:61. doi: 10.1186/s13049-014-0061-3 25385311 PMC4234828

[11] Jensen B, Vardinghus-Nielsen H, Mills EHA, Møller AL, Gnesin F, Zylyftari N, et al. “Like a rainy weather inside of me”: qualitative content analysis of telephone consultations concerning back pain preceding out-of-hospital cardiac arrest. Int Emerg Nurs. 2022;64:101200. doi: 10.1016/j.ienj.2022.101200 35926318

[12] Møller TP, Ersbøll AK, Tolstrup JS, Østergaard D, Viereck S, Overton J, et al. Why and when citizens call for emergency help: an observational study of 211,193 medical emergency calls. Scand J Trauma Resusc Emerg Med. 2015;23:88. doi: 10.1186/s13049-015-0169-0 26530307 PMC4632270

[13] Forslund K, Kihlgren M, Ostman I, Sørlie V. Patients with acute chest pain - experiences of emergency calls and pre-hospital care. J Telemed Telecare. 2005;11(7):361–7. doi: 10.1258/135763305774472006 16238838

[14] Richards SH, Pound P, Dickens A, Greco M, Campbell JL. Exploring users’ experiences of accessing out-of-hours primary medical care services. Qual Saf Health Care. 2007;16(6):469–77. doi: 10.1136/qshc.2006.021501 18055893 PMC2653185

[15] Jepsen K, Rooth K, Lindström V. Parents’ experiences of the caring encounter in the ambulance service-A qualitative study. J Clin Nurs. 2019;28(19–20):3660–8. doi: 10.1111/jocn.14964 31188508

[16] Nord-Ljungquist H, Engström Å, Fridlund B, Elmqvist C. Lone and lonely in a double ambivalence situation as experienced by callers while waiting for the ambulance in a rural environment. Scand J Caring Sci. 2020;34(3):566–74. doi: 10.1111/scs.12767 31614024

[17] Forslund K, Quell R, Sørlie V. Acute chest pain emergencies - spouses’ prehospital experiences. Int Emerg Nurs. 2008;16(4):233–40. doi: 10.1016/j.ienj.2008.07.001 18929341

[18] Larsson G, Dagerhem A, Wihlborg J, Rantala A. Satisfaction among non-conveyed patients and significant others when discharged at the scene by the ambulance service: an exploratory cross-sectional survey. BMC Emerg Med. 2022;22(1):100. doi: 10.1186/s12873-022-00659-9 35672702 PMC9171931

[19] King R, Oprescu F, Lord B, Flanagan B. Patient experience of non-conveyance following emergency ambulance service response: a scoping review of the literature. Australas Emerg Care. 2021;24(3):210–23. doi: 10.1016/j.auec.2020.08.006 32943367

[20] van Doorn SCM, Verhalle RC, Ebben RHA, Frost DM, Vloet LCM, de Brouwer CPM. The experience of non-conveyance following emergency medical service triage from the perspective of patients and their relatives: A qualitative study. Int Emerg Nurs. 2021;54:100952. doi: 10.1016/j.ienj.2020.100952 33383408

[21] Forsell L, Forsberg A, Kisch A, Rantala A. Inequalities and short-term outcome among patients assessed as non-urgent in a Swedish ambulance service setting. Int Emerg Nurs. 2021;57:101018. doi: 10.1016/j.ienj.2021.101018 34147876

[22] von Elm E, Altman DG, Egger M, Pocock SJ, Gøtzsche PC, Vandenbroucke JP, et al. The Strengthening the Reporting of Observational Studies in Epidemiology (STROBE) Statement: guidelines for reporting observational studies. Int J Surg. 2014;12(12):1495–9. doi: 10.1016/j.ijsu.2014.07.013 25046131

[23] Laerdal. The Leardal Foundation for Acute medicine; Swedish Index to emergency medical assistance. Stavanger: Leardal A/S; 2007.

[24] Holmström I, Dall’Alba G. “Carer and gatekeeper” - conflicting demands in nurses’ experiences of telephone advisory services. Scand J Caring Sci. 2002;16(2):142–8. doi: 10.1046/j.1471-6712.2002.00075.x 12000667

[25] Gamst-Jensen H, Frischknecht Christensen E, Lippert F, Folke F, Egerod I, Huibers L, et al. Self-rated worry is associated with hospital admission in out-of-hours telephone triage - a prospective cohort study. Scand J Trauma Resusc Emerg Med. 2020;28(1):53. doi: 10.1186/s13049-020-00743-8 32522240 PMC7288501

[26] Gamst-Jensen H, Huibers L, Pedersen K, Christensen EF, Ersbøll AK, Lippert FK, et al. Self-rated worry in acute care telephone triage: a mixed-methods study. Br J Gen Pract. 2018;68(668):e197–203. doi: 10.3399/bjgp18X695021 29440015 PMC5819985

[27] O’connor P, O’malley R, Lambe K, Byrne D, Lydon S. How safe is prehospital care? A systematic review. Int J Qual Health Care. 2021;33(4):mzab138. doi: 10.1093/intqhc/mzab138 34623421 PMC8547145

[28] Höglund E, Schröder A, Andersson-Hagiwara M, Möller M, Ohlsson-Nevo E. Outcomes in patients not conveyed by emergency medical services (EMS): a one-year prospective study. Scand J Trauma Resusc Emerg Med. 2022;30(1):40. doi: 10.1186/s13049-022-01023-3 35698086 PMC9195370

[29] Lederman J, Lindström V, Elmqvist C, Löfvenmark C, Ljunggren G, Djärv T. Non-conveyance of older adult patients and association with subsequent clinical and adverse events after initial assessment by ambulance clinicians: a cohort analysis. BMC Emerg Med. 2021;21(1):154. doi: 10.1186/s12873-021-00548-7 34895152 PMC8666056

[30] Victor CR, Peacock JL, Chazot C, Walsh S, Holmes D. Who calls 999 and why? A survey of the emergency workload of the London Ambulance Service. J Accid Emerg Med. 1999;16(3):174–8. doi: 10.1136/emj.16.3.174 10353041 PMC1343328

